# Comparative Analysis of *Lacinutrix* Genomes and Their Association with Bacterial Habitat

**DOI:** 10.1371/journal.pone.0148889

**Published:** 2016-02-16

**Authors:** Yung Mi Lee, Mi-Kyeong Kim, Do Hwan Ahn, Han-Woo Kim, Hyun Park, Seung Chul Shin

**Affiliations:** 1 Korea Polar Research Institute, 26 Songdomirae-ro, Yeonsu-gu, Incheon, 406–840, Korea; 2 University of Science & Technology, Yuseong-gu, Daejeon, 305–333, Korea; Massey University, NEW ZEALAND

## Abstract

The genus *Lacinutrix*, which belongs to the family *Flavobacteriaceae*, consists of seven bacterial species that were mainly isolated from marine life and sediments. As most bacteria in the family *Flavobacteriaceae* favor aerobic conditions, the seven bacterial species in the genus *Lacinutrix* also showed aerobic growth. We selected four monophyletic bacterial species living in a polar environment. Two of these species were isolated from sediment and two types were isolated from algae. In a comparative analysis, we investigated how these different environments were related to genomic features of these four species in the genus *Lacinutrix*. We found that the gene sets for glycolysis, the Krebs cycle, and oxidative phosphorylation were conserved in these four type strains. However, the presence of nitrous oxide reductase for denitrification and the absence of essential components related to thiamin biosynthesis for aerobic respiration were only found in isolates from sediment. Elevated bacterial metabolism on the surface of marine sediments might limit the oxygen penetration into sediment, and such an environment might affect the genomes of bacteria isolated from these habitats.

## Introduction

The genus *Lacinutrix* was established with the species *Lacinutrix copepodicola* and was determined to belong to family *Flavobacteriaceae* [1]. Seven bacterial species in this genus have been reported to date [1–4]. All of these species were isolated from marine environments and five were isolated from polar regions. *L*. *copepodicola* was isolated from a copepod found in a lake in Antarctica, *L*. *algicola* AKS293^T^ and *L*. *mariniflava* AKS432^T^ were isolated from a marine red algae at Marian Cove on King George Island, Antarctica, *L*. *himadriensis* E4-9a^T^ was isolated from marine sediment from Kongsfjorden Svalbard in the Arctic Ocean, and *L*. *jangbogonensis* PAMC 27137^T^ was isolated from marine sediment from the Ross Sea, Antarctica. *L*. *undariae* was isolated from a brown algae reservoir in the South Sea, South Korea [5] and *L*. *venerupis* was isolated from clams (*Venerupis decussata* and *Venerupis philippinarum*) in Galicia (NW Spain) [6]. The seven species are psychrophilic, growing at 4–25°C [1–4] and grow aerobically, regardless of habitat.

Two of the seven species of *Lacinutrix* were isolated from sediment samples, and members of this genus were found to be the main cultivable protease-producing bacteria in sediment of Maxwell Bay, King George Island, Antarctica [7]. Because oxygen is not expected to be present below a few centimeters of sediment [8], this habitat might be considered unfavorable for *Lacinutrix* to grow aerobically. Recently, the genome of PAMC 27137^T^ was reported to be approximately four megabases [9], but details on genomic features were not included; hence, no associations between genome content and habitat could be made.

A comparative genomic study on species from the same genus might provide clues to understand bacterial adaptation and evolution in their environments. In this study, we investigated by comparative genetic analyses how *Lacinutrix* species may have adapted to their environments. Here, we also provide a detailed description of genome characteristics of this genus.

## Material and Methods

### Strains

Strains *Lacinutrix jangbogonensis* PAMC 27137^T^ and *L*. *himadriensis* E4-9a^T^ (KCTC 23612^T^), both isolated from sediments, were obtained from the Polar and Alpine Microbial Collection (PAMC) of Korea Polar Research Institute and the Korean Collection for Type Cultures (KCTC), respectively. Strains *L*. *mariniflava* AKS432^T^ (JCM 13824) and *L*. *algicola* AKS293^T^ (JCM 13825), isolated from marine algae were obtained from the Japan Collection of Microorganisms (JCM). *Lacinutrix* species were routinely cultured on Marine Agar media at 10°C.

### Phylogenetic analysis

The 16S rRNA gene sequences retrieved from whole genome sequencing were compared with those of all bacterial strains in the EzTaxon-e database [10]. The sequences were aligned with those of bacterial strains that were phylogenetically related by using Ribosomal Database Project II [11], and the alignments was checked manually using the PHYDIT (ver. 3.2) program (http://plaza.snu.ac.kr/~jchun/phydit/). A phylogenetic tree was constructed by the neighbor-joining method [12] using MEGA version 6 [13]. The robustness of the tree topology was assessed by bootstrap analysis based on 1,000 replicates.

### Genome sequencing and assembly

The genome sequence of PAMC 27137^T^ (JSWF01000001-JSWF01000046) was obtained from GenBank database [9]. For AKS432^T^ and AKS293^T^, adding to extended Sanger-like reads from a 454 GS FLX+ system (Roche Diagnostics, Branford, CT) in a previous study [4], a 8-kb paired-end library was constructed and sequenced using a 454 GS FLX Titanium system (Roche Diagnostics, Branford, CT). The E4-9a^T^ genome was sequenced (2×300 bp) using an Illumina MiSeq (Illumina, San Diego, CA). All 454 and Illumina sequencing reads were converted to an FRG file format using sffToCA and fastqToCA, respectively, for assembly, which was performed using a Celera Assembler (ver. 7.0) with the parameters overlapper = ovl, unitigger = bogart, merSize = 22, and doOverlapBasedTrimming = 1 [14].

### Genome annotation and assembly validation

Genomes were annotated with Rapid Annotation using Subsystems Technology (RAST) [15], and Gene Ontology (GO) terms were assigned using Blast2GO [16]. For the analysis of gene presence/absence, sequencing reads were re-mapped onto the assemblies using Bowtie 2 (Ver. 2.2.6) [17], and unmapped read were identified using BLASTx against the target protein database. The amino acid sequences of the presence gene were also identified using tBLASTx against the target draft genome sequence.

### Cluster analysis of orthologs

We identified orthologous groups using OrthoMCL (Ver. 2.0.5) [18], which generated a graphical representation of sequence relationships that was then divided into subgraphs using the Markov Clustering Algorithm (MCL). OrthoMCL pairs sequences using an all-versus-all BLAST, and then clusters the pairs to orthologous groups using the MCL program. We used the amino acid sequences annotated from RAST and executed OrthoMCL with the standard parameters and options (a percent match cutoff = 50 and the E-value exponent cutoff = 10^−5^) for all steps.

### Genomic alignment

The four sequenced genomes were aligned to the assembled genome scaffolds using NUCmer (Ver. 3.07) with default settings [19]. Mummerplot (Ver. 3.5) was used with the NUCmer delta file as input to generate the plots.

### Gene enrichment analysis

We used AgriGO [20], a web-based tool for gene ontology analysis, with a significance level of P < 0.05, and complete hierarchies of GO terms for each gene to identify enriched GOs.

### Data access

The whole genome shotgun projects have been deposited in DDBJ/EMBL/GenBank under assession number LIQI00000000 (*L*. *himadriensis* E4-9a^T^), LIQG00000000 (*L*. *mariniflava* AKS432^T^), and LIQH00000000 (*L*. *algicola* AKS293^T^).

## Results and Discussion

### Genome sequencing, gene annotation, and synteny

**Fig 1:**
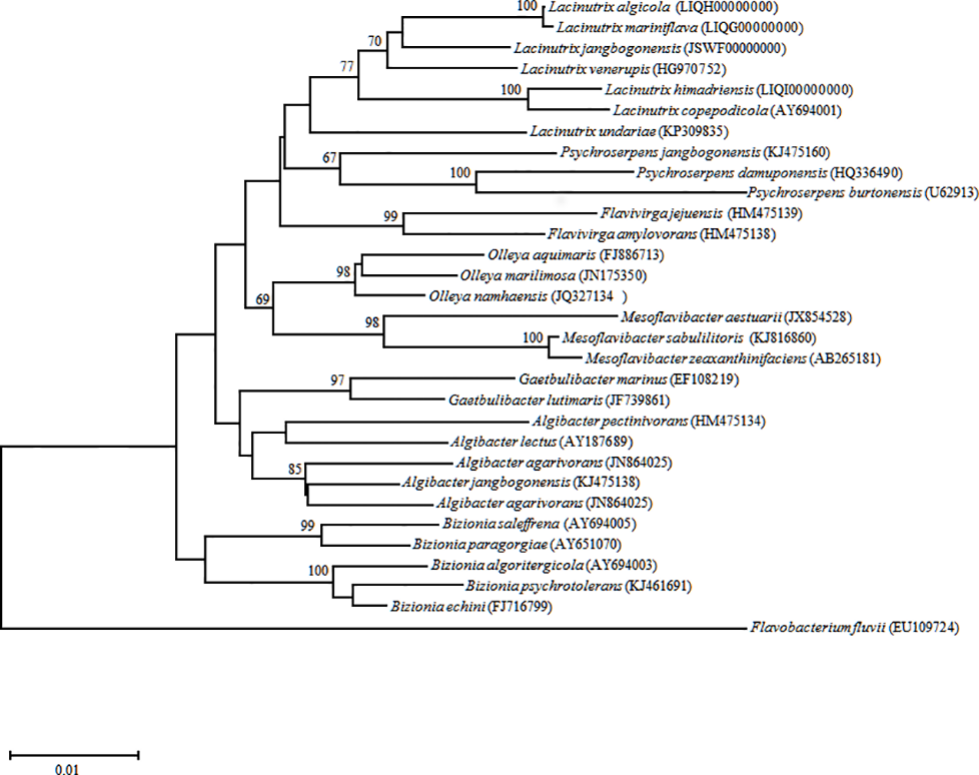
Neighbor-joining phylogenetic tree based on 16S rRNA gene sequences showing the relationships of *Lacinutrix* species and other closely related members of the family *Flavobacteriaceae*. Bootstrap values (>60%) based on 1,000 resamplings are shown above the nodes. Bar, 0.01 substitution per nucleotide position. *Flavobacterium fluvii* H7 was used as the outgroup.

We previously reported the draft genome sequence of PAMC 27137^T^ [9]. Here, to better understand the genus *Lacinutrix* and its environmental adaptations, we selected three of the closest type strains (AKS293^T^, AKS432^T^, and E4-9a^T^) for a comparative genetic analysis (Fig 1). PAMC 27137^T^ and E4-9a^T^ were isolated from marine sediments in the Southern and Artic Oceans, respectively, and the other two strains were isolated from algae in the Southern Ocean. To determine adaptations to different environments in polar regions, *L*. *copepidicola*, which alone was isolated from a copepod, was excluded from analysis. Bacterial species isolated from non-polar regions were also excluded.

**Fig 2:**
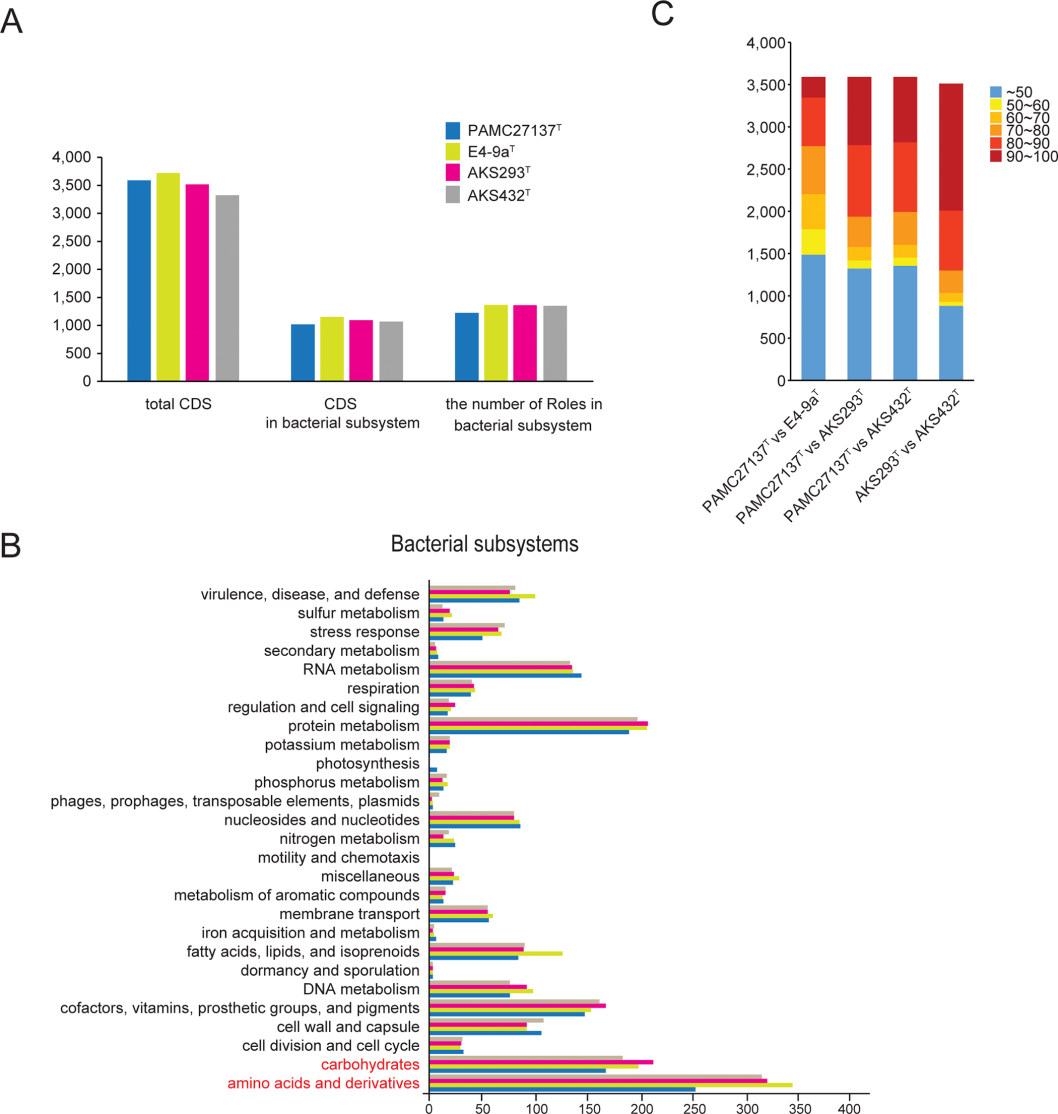
General genomic features of the four *Lacinutrix* species. (A) The number of coding DNA sequences (CDS) in each bacterial subsystem and the number of roles they perform, (B) the number of genes by bacterial subsystem (shown in red text) are the bacterial subsystems that have a relatively small number of genes in PAMC 27137^T^ compared to that in other *Lacinutrix*. (C) The similarity of CDS among the four type strains was calculated using BLASTp

We performed genomic sequencing with an average coverage of more than 24 folds. The number of scaffolds ranged from 12 to 30, the number of contigs ranged from 32 to 46, and the N50 contig lengths ranged from 181 kb to 374 kb (Table 1). Each type strain genome comprised between 3,321 and 3,718 genes, and the total coding region was about 84–89% of the full draft genome sequence (Table 2). The draft genome sequence of AKS293^T^ was smaller than the others and comprised a smaller number of coding DNA sequences (CDS). However, the number of genes, which were included in functional categories of RAST, was similar to that of the two other type strains (AKS432^T^ and E4-9a^T^) (Fig 2A). PAMC 27137^T^, however, was found to contain only 1,658 genes, representing the smallest number of genes in functional categories among the four type strains examined, and showed a relatively small number of genes in the categories “amino acids and derivatives” and “carbohydrates” (Fig 2B). We investigated whether the genes involved in amino acid biosynthesis and carbohydrate metabolism were conserved in PAMC 27137^T^, and the losses of seven genes related to biosynthesis of L-arginine from N-acetyl-L-glutamate through L-ornithine and five genes related to biosynthesis of starch were identified.

**Table 1:** *Lacinutrix* genome assembly statistics.

	PAMC 27137^T^	E4-9a^T^	AKS293^T^	AKS432^T^
**Total scaffolds**	14	30	12	19
**Total bases in scaffolds**	4,017,559	4,166,348	3,974,017	3,661,498
**Scaffolds N50 (bases)**	907,640	351,089	3,961,528	619,104
**Scaffolds max (bases)**	1,902,948	990,725	3,961,528	1,095,301
**Total contigs**	46	32	32	46
**Total bases in contigs**	4,017,559	4,166,348	3,974,017	3,661,498
**Contigs N50 (bases)**	181,213	351,089	374,262	204,394
**Contigs max (bases)**	370,182	990,725	1,246,183	449,564
**Total reads**	223,923	3,918,097	326,784	218,771
^**a**^**Single reads**	80,831		101,644	75,806
^**b**^**8-kb paired-end reads**	143,902		225,140	142,965
^**c**^**500-bp paired-end reads**		3,918,097		
**Fold-coverage**	24.3	177.35	39.57	26.49
**Mapping-rate (%)**	98.9	99.5	98.6	97.7
**G+C content (%)**	30.61	32.28	29.51	29.81

^a^ reads generated from a 454 GS FLX+ system

^b^ reads generated from a 454 GS FLX Titanium system

^c^ reads generated from an Illumina MiSeq

**Table 2:** Draft genome annotation statistics.

	PAMC 27137^T^	E4-9a^T^	AKS293^T^	AKS432^T^
**CDS**	3590	3718	3513	3321
**tRNA**	38	35	38	36
**rRNA**	8	4	9	13
**CDS (length)**	3,498,419	3,693,211	3,507,543	3,238,309
**CDS (%)**	87.078	88.644	84.187	88.442

To compare proteins encoded in the genomes of the four type strains, similarities of their predicted sequences were calculated using BLASTp (Fig 2C). We found that the amino acid identity between PAMC 27137^T^ and E4-9a^T^ was the lowest among the compared sequences, and that the highest protein identity was between AKS293^T^ and AKS432^T^. The number of protein sequences with >90% identity between AKS293^T^ and AKS432^T^ was 1,502, which is approximately 42.8% of the total amino acid sequence. In contrast, the number of protein sequences with >90% identity between PAMC 27137^T^ and E4-9a^T^ was approximately 6.7% of the total protein coding sequence.

Next, we compared the draft genomes of three strains isolated from the Southern Ocean, and the result, based on the nucleotide sequences, showed that these three strains have the greatest synteny, although many translocations and inversions were identified (S1 Fig). However, a comparison with the draft genome sequence of E4-9a^T^ from the Arctic Ocean showed reduced linearity. Among the four *Lacinutrix* strains, E4-9a^T^ showed the lowest sequence similarity and synteny with other species.

### Central metabolism

**Fig 3:**
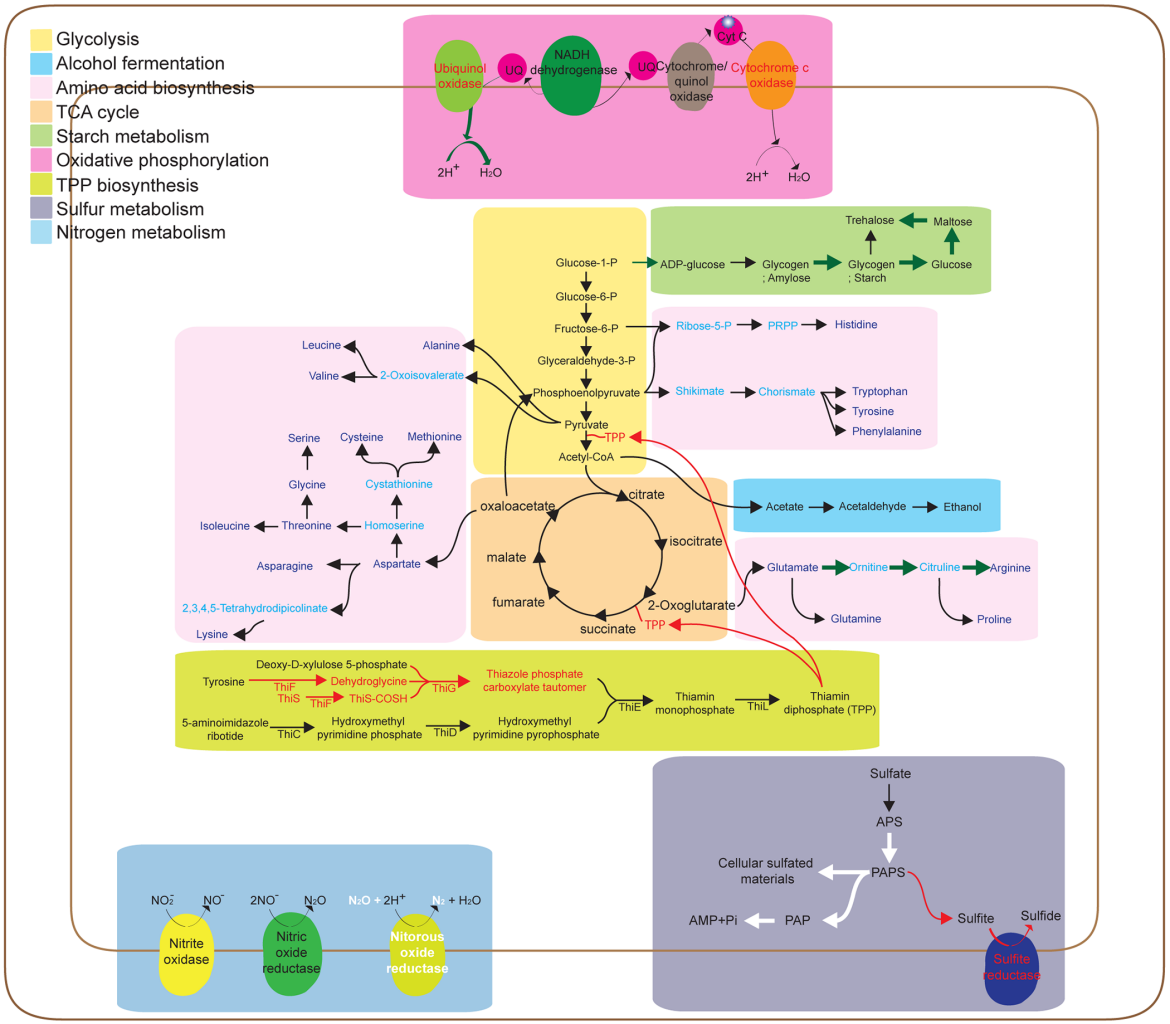
An overview of the predicted central metabolism and respiration pathways of the four type strains. Red arrows and text show genes absent in bacteria isolated from sediments. White arrows indicate the genes gained by bacteria isolated from sediment. Green arrows denote genes lost from PAMC 27137^T^. Amino acids are shown in violet

We deduced a central metabolic pathway from the results of the KEGG pathway analysis. Though PAMC 27137^T^ showed the smallest number of gene in functional categories, the components of glycolysis, the citric acid cycle, and oxidative phosphorylation were well conserved among the four type strains. This indicates that the strains in this study could convert glucose into two pyruvate molecules, and that pyruvate may be converted into adenosine triphosphate (ATP) through the citric acid cycle and oxidative phosphorylation (Fig 3). The four type strains was found to be capable of producing ethanol and carbon dioxide from acetyl-CoA and synthesizing all 20 amino acids, with the exception of PAMC 27137^T^, which lacked the genes necessary to produce arginine from glutamate. Genes encoding acetylornithine deacetylase, ornithine carbamoyltransferase, argininosuccinate synthase, and argininosuccinate lyase were absent in PAMC 27137^T^. Glucose could be converted to starch by all strains except PAMC 27137^T^, which lacked the starch synthase gene.

### Cold shock and heat shock proteins

In the four type strains, we found the multiple genes for cold shock protein A (CspA), a single gene for cold shock protein G (CspG), and several genes for heat shock proteins (GroEL, GroES, DnaK, and DnaJ) (S1 Table). CspA is the major cold shock protein in *Escherichia coli*; mutants that do not express CspA cannot grow at either 15°C or 37°C [21, 22], and chaperonins from cold adapted bacteria facilitate the growth of *E*. *coli* at low temperatures [23]. We could not determine whether this gene is related to the cold adaptation, but cold shock proteins and heat shock proteins might help *Lacinutrix* species to survive in low temperatures.

### Orthologous genes and adaptation

**Fig 4:**
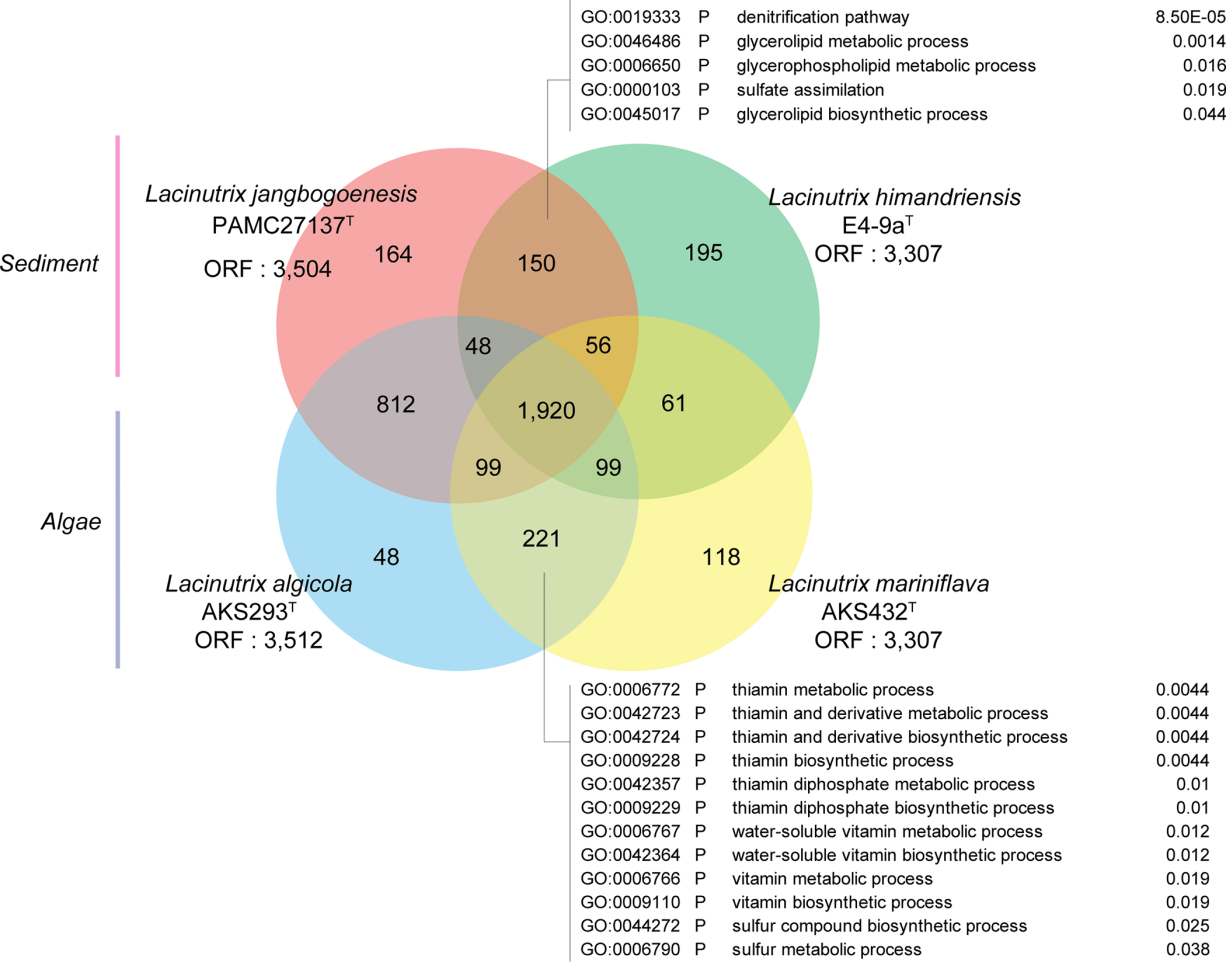
Venn diagram showing the overlap in the gene families of the four type strains. In strains from sediment and algae, 5 and 12 GO terms were significantly enriched, respectively

To explore the evolutionary dynamics of the four type strains, we first investigated orthologous groups using OrthoMCL [18], and we identified 1,920 orthologous groups among the type strains (Fig 4). To determine which functional gene categories were associated with habitat (sediments or algae), we focused on the genes shared between strains from a common environment and performed a GO enrichment analysis using 150 selected genes shared between the two strains from sediment. Three GO terms (denitrification pathway [GO:0019333], sulfate assimilation [GO:0000103], and glycerophospholipid metabolic process [GO:0006650]) were significant in the enrichment analysis. Four genes in the GO term denitrification pathway comprise encode nitrous oxide reductase, which catalyzes the reduction of nitrous oxide to dinitrogen in the final step of bacterial denitrification [24, 25]. In marine sediment, denitrification is an important function of bacteria [26], and this analysis suggests that the type strains living in sediments might function as denitrifiers contributing to the global ocean nitrogen budget. The presence of genes encoding adenylyl-sulfate (APS) kinase and putative adenosine 3',5'-bisphosphate (PAP) phosphatase also made sulfur assimilation a significant GO term. The strains isolated from sediment could convert APS to 3´,5´-bisphosphate adenylyl sulfate (PAPS) with APS kinase; the sulfate of PAPS could be used as the source of cellular sulfated materials through the putative sulfotransferase, and the resulting PAP could be converted into adenosine monophosphate and phosphate by PAP phosphatase (Fig 3) [27]. However, the strains isolated from algae have a PAPS reductase and sulfite reductase without an APS kinase; therefore, assimilation of sulfate molecules might be impossible. However, PAPS might be converted to sulfide (Fig 3). These data suggest that strains isolated from sediment might have developed the ability to synthesize sulfur-containing cell components to reduce and assimilate sulfate. In contrast, strains isolated from algae have evolved to reduce sulfate to sulfide.

Another noteworthy GO term in the strains from both habitats was biosynthesis of thiamine phosphate. Fourteen GO terms were found to be significant in the GO enrichment analysis using 221 orthologs that were common to the strains isolated from algae. Among these, 12 GO terms were related to thiamin biosynthetic process and two GO terms were related to the sulfur metabolic process. However, all 14 of these GO terms converged into the biosynthesis of thiamine in the GO hierarchy. The synthesis of thiamine monophosphate could be catalyzed by thiamine phosphate synthase (ThiE) with hydroxymethyl pyrimidine pyrophosphate and thiazole phosphate carboxylate tautomer. However, the type strains of *Lacinutrix* found in sediments were previously shown to have lost the adenylyltransferase (ThiF), sulfur carrier protein (ThiS), and thiazole synthase (ThiG) functions in the biosynthetic pathway of thiazole phosphate carboxylate tautomer (Fig 3) [28]. Thiamine pyrophosphate (TPP) is an essential cofactor for most organisms; it is an important coenzyme that catalyzes the transfer of two-carbon units, and is essential in both glycolysis and the citric acid cycle as a cofactor for pyruvate dehydrogenase and 2-oxoglutarate dehydrogenase, respectively [29]. Therefore, thiamine is indispensable for aerobic processing of glucose, which is the source used to generate most adenosine triphosphate (ATP). The loss of essential components for TPP biosynthesis suggests that thiazole phosphate carboxylate tautomer or thiamine might be abundant in sediment habitats [30] or that sediment environments might be partially microaerobic or anaerobic [8]. In sediments, rates of microbial respiration are high and oxygen penetrates only millimeters to centimeters in depth [8, 31]. Thus, the *Lacinutrix* strains in sediment might have lost the genes encoding the products involved in the synthesis of thiazole phosphate carboxylate tautomer.

In this study, we showed that, despite the geographic distance between locations where these strains were isolated, a few bacterial strains of the genus *Lacinutrix* isolated from similar habitats shared critical genome components. Though the identity of the amino acid sequences of strains isolated from sediment was lower than strains from algae, the results of the GO enrichment analyses showed that the patterns of gene loss or gene gain were similar. In marine sediments, high rates of microbial respiration hinder oxygen penetration, making the local environment microaerobic or anaerobic [31]. Though we did not measure the oxygen in sediments from which *Lacinutrix* strains in this study were isolated, the type strains isolated from sediments might have acquired nitrous oxide reductase for denitrification as an additional tool for respiration under low oxygen or anoxic condition [26], and might have adapted to obtain intermediates in TPP biosynthesis, which are essential for aerobic respiration, from environment instead of *de novo* TPP biosynthesis.

## Supporting Information

S1 Fig Synteny analyses of the draft genomes of *Lacinutrix* strains based on nucleotide sequences. (DOCX)

S1 Table Cold shock and heat shock protein genes. (DOCX)
