# Supplementary figures and images for: Comparative Analysis of Lacinutrix Genomes and Their Association with Bacterial Habitat
Source: PLoS One. 2016 Feb 16;11(2):e0148889. doi: 10.1371/journal.pone.0148889 (PMC4755562; doi:10.1371/journal.pone.0148889)

**S1 Fig.**


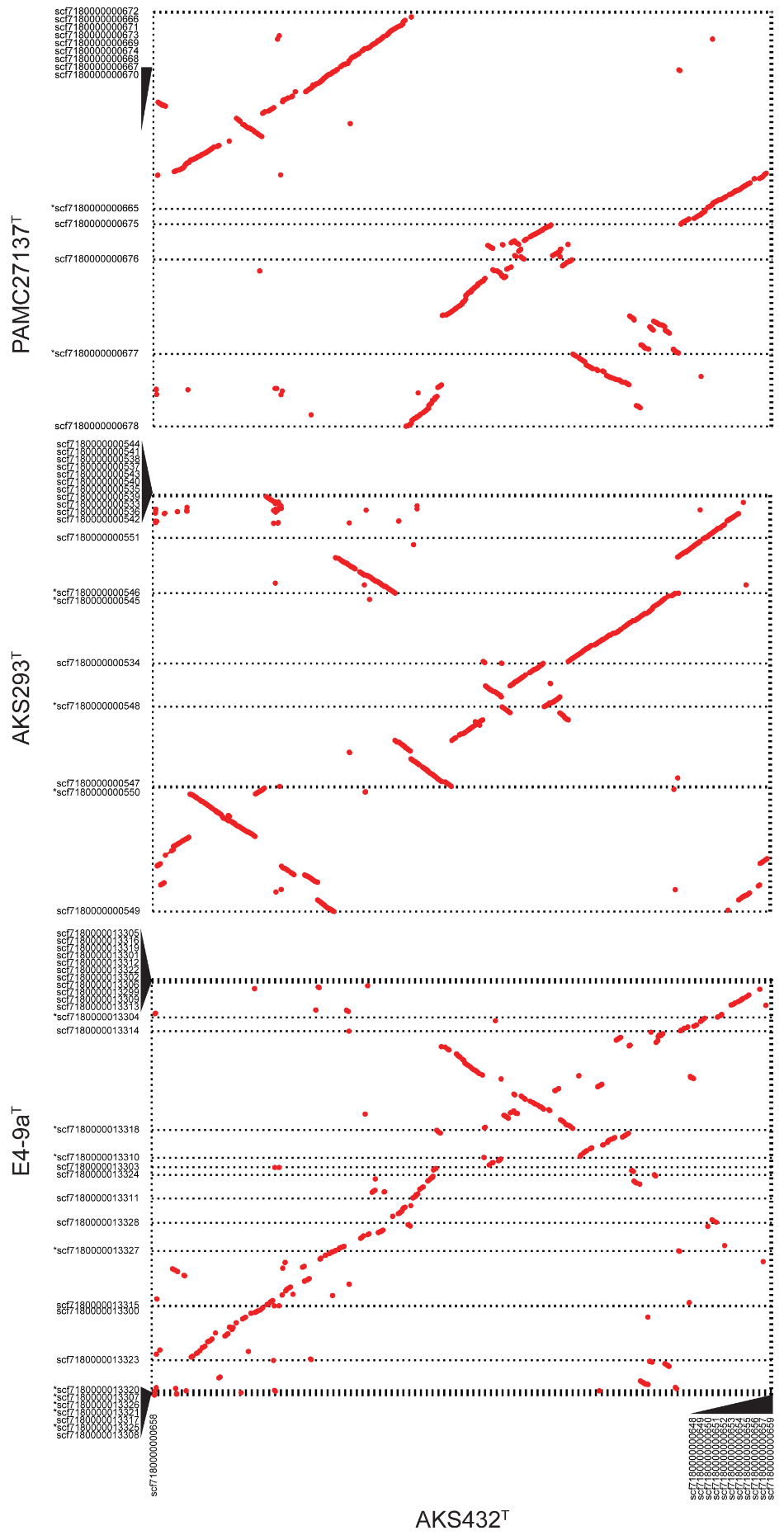

Supplement: S1 Fig — (DOCX) [file pone.0148889.s001.docx]
