# Supplementary material for: Comparative Analysis of Lacinutrix Genomes and Their Association with Bacterial Habitat
Source: PLoS One. 2016 Feb 16;11(2):e0148889. doi: 10.1371/journal.pone.0148889 (PMC4755562; doi:10.1371/journal.pone.0148889)
Supplement: S1 Table — (DOCX) [file pone.0148889.s002.docx]

**S1 Table.**

|  | PAMC 27137^T^ | E4-9a^T^ | AKS293^T^ | AKS432^T^ |
| --- | --- | --- | --- | --- |
| cspA | 2 | 4 | 5 | 4 |
| cspG | 1 | 1 | 1 | 1 |
| groEL | 1 | 1 | 1 | 1 |
| groES | 1 | 1 | 1 | 1 |
| dnaK  dnaJ | 2  1 | 2  2 | 1  1 | 1  1 |
|  |  | |  |  |
